# Post-COVID-19 interstitial lung disease: Insights from a machine learning radiographic model

**DOI:** 10.3389/fmed.2022.1083264

**Published:** 2023-01-17

**Authors:** Theodoros Karampitsakos, Vasilina Sotiropoulou, Matthaios Katsaras, Panagiota Tsiri, Vasiliki E. Georgakopoulou, Ilias C. Papanikolaou, Eleni Bibaki, Ioannis Tomos, Irini Lambiri, Ourania Papaioannou, Eirini Zarkadi, Emmanouil Antonakis, Aggeliki Pandi, Elli Malakounidou, Fotios Sampsonas, Sotiria Makrodimitri, Serafeim Chrysikos, Georgios Hillas, Katerina Dimakou, Nikolaos Tzanakis, Nikolaos V. Sipsas, Katerina Antoniou, Argyris Tzouvelekis

**Affiliations:** ^1^Department of Respiratory Medicine, University General Hospital of Patras, Patras, Greece; ^2^Department of Infectious Diseases-COVID-19 Unit, Laiko General Hospital, Athens, Greece; ^3^Department of Respiratory Medicine, Corfu General Hospital, Corfu, Greece; ^4^Laboratory of Molecular and Cellular Pneumonology, Department of Thoracic Medicine, Medical School, University of Crete, Heraklion, Greece; ^5^5th Department of Respiratory Medicine, Hospital for Thoracic Diseases, ‘SOTIRIA’, Athens, Greece; ^6^Medical School, National and Kapodistrian University of Athens, Zografou, Greece

**Keywords:** post-COVID-19, long COVID, interstitial lung disease, antifibrotics, machine learning

## Abstract

**Introduction:**

Post-acute sequelae of COVID-19 seem to be an emerging global crisis. Machine learning radiographic models have great potential for meticulous evaluation of post-COVID-19 interstitial lung disease (ILD).

**Methods:**

In this multicenter, retrospective study, we included consecutive patients that had been evaluated 3 months following severe acute respiratory syndrome coronavirus 2 infection between 01/02/2021 and 12/5/2022. High-resolution computed tomography was evaluated through Imbio Lung Texture Analysis 2.1.

**Results:**

Two hundred thirty-two (*n* = 232) patients were analyzed. FVC% predicted was ≥80, between 60 and 79 and <60 in 74.2% (*n* = 172), 21.1% (*n* = 49), and 4.7% (*n* = 11) of the cohort, respectively. DLCO% predicted was ≥80, between 60 and 79 and <60 in 69.4% (*n* = 161), 15.5% (*n* = 36), and 15.1% (*n* = 35), respectively. Extent of ground glass opacities was ≥30% in 4.3% of patients (*n* = 10), between 5 and 29% in 48.7% of patients (*n* = 113) and <5% in 47.0% of patients (*n* = 109). The extent of reticulation was ≥30%, 5–29% and <5% in 1.3% (*n* = 3), 24.1% (*n* = 56), and 74.6% (*n* = 173) of the cohort, respectively. Patients (*n* = 13, 5.6%) with fibrotic lung disease and persistent functional impairment at the 6-month follow-up received antifibrotics and presented with an absolute change of +10.3 (*p* = 0.01) and +14.6 (*p* = 0.01) in FVC% predicted at 3 and 6 months after the initiation of antifibrotic.

**Conclusion:**

Post-COVID-19-ILD represents an emerging entity. A substantial minority of patients presents with fibrotic lung disease and might experience benefit from antifibrotic initiation at the time point that fibrotic-like changes are “immature.” Machine learning radiographic models could be of major significance for accurate radiographic evaluation and subsequently for the guidance of therapeutic approaches.

## Introduction

The global impact of the ongoing coronavirus disease 2019 (COVID-19) pandemic has been unparalleled. The disease course is variable, manifesting from asymptomatic to fatal forms, and long-term complications could have further devastating effects (1–3). Emerging evidence indicate that a substantial proportion of infected individuals may experience prolonged symptoms lasting for more than 6 months (4–6).

The National Institute for Health and Care Excellence (NICE) and the Centers for Disease Control and prevention (CDC) define long COVID as symptoms and sequalae that persist or develop after the 4-week acute phase of COVID-19 and that cannot be explained by an alternative diagnosis (7, 8). This term includes ongoing symptomatic COVID-19, which encompasses manifestations from 4 to 12 weeks post-infection, and post-COVID-19 syndrome, which refers to symptoms/clinical signs beyond 12 weeks following infection (8). The pathogenic process of this recently reported condition has not been elucidated; yet potential contributing mechanisms include viral toxicity inducing ACE2 downregulation (due to cellular internalization) thus leading to a pro-fibrotic microenvironment as well as immune dysregulation with emergence of autoimmunity phenomena leading to persistent inflammatory damage (9). Post-COVID clinical manifestations are multisystemic and the lasting symptom burden may lead to severe functional limitation and decrement in quality of life (9). Pulmonary sequalae range in a wide clinical, physiologic, and imaging spectrum and have been related to the severity of acute illness (9–11). The most commonly reported pulmonary manifestations are diffusion capacity decline, restrictive pattern with regards to functional impairment and ground glass opacities with or without fibrotic lesions in the context of radiographic signs (12, 13).

Meticulous radiographic evaluation might have a cardinal role for the guidance of therapeutic approaches in this new entity with unknown long-term effects. Toward this direction, we performed patients’ radiographic evaluation through a validated machine learning software system, denominated Imbio Lung Texture Analysis (14). Machine learning represents a subgroup of artificial intelligence. In this setting, computers extract patterns from appropriately classified input data and accordingly generate labels for new, unknown data (15). Machine learning and its subset named deep learning have demonstrated great potential in multiple medical imaging classification tasks including prediction of mortality in Idiopathic Pulmonary Fibrosis (16–20).

This multicenter study aimed to present functional and radiographic features of patients with post-COVID-19-interstitial lung disease (ILD), in a quantitative, precise and unbiased fashion using cutting-edge technology, highlighting the importance of screening patients with long-COVID-19 clinical, and functional and radiological impairment.

## Materials and methods

### Trial design and oversight

In this multicenter, investigator-initiated, retrospective, observational cohort study, we included consecutive patients that had been evaluated 3 months following severe acute respiratory syndrome coronavirus 2 (SARS-CoV-2) infection between 01/02/2021 and 12/05/2022. Trial sites were five referral ILD centers in Greece. Patients with positive polymerase chain reaction test for SARS-CoV-2, treated both in outpatient and inpatient setting were included. Patients with less than 18 years of age were excluded from the analysis.

We recorded PaO_2_/FiO_2_ during hospitalization, demographics, comorbidities, as well as forced vital capacity (FVC), forced expiratory volume in 1 s (FEV1), diffusing capacity of the lung for carbon monoxide (DLCO) and High-Resolution Computed Tomography (HRCT) findings 3 months following COVID-19 infection.

High-Resolution Computed Tomography scans were evaluated through the validated machine learning software, named Imbio Lung Texture Analysis version 2.1 (14). Imbio Lung Texture Analysis had the following technical requirements for HRCT analysis: Minimal movement and acceptable position, slice thickness <2.0 mm, revolution time <1 s, pixel spacing <2.0 mm, and slice spacing <2.0 mm. Subsequently, Imbio Lung Texture Analysis version 2.1 provided a report with% of each lobe and % of lung as total that was characterized as: Normal, ground glass, reticular, honeycombing or hyperlucent. A succinct rating scale for % disease extent was applied, providing 1% step evaluation for disease extent.

The trial was conducted in accordance with the International Conference on Harmonization E6 guidelines for Good Clinical Practice, the Declaration of Helsinki and the local regulations. Management was based on a common algorithm. Radiographic findings of the patients were initially meticulously evaluated and split into two groups: (1) Fibrotic-like lesions and (2) inflammatory abnormalities only. Subsequently, patients were further divided based on their functional status. Retrospective data collection and analysis was approved by our institutional review board (protocol number: 16574/29-6-2022).

### Outcome measures

Outcome measures included: (1) The frequency of functional impairment as indicated by decline in FVC% predicted, FEV1% predicted or DLCO% predicted, (2) the frequency of specific radiographic findings following Imbio Lung Texture Analysis, and (3) the effectiveness of antifibrotics as indicated by change in FVC% predicted, DLCO% predicted at 3, 6, and 12 months following treatment initiation and changes in HRCT.

### Statistical analysis

Continuous data were reported as mean ± standard deviation (SD) or medians with 95% Confidence Interval (95% CI) following Kolmogorov–Smirnov test for normality. Frequency tables and graphs were drawn. ANOVA repeated measures was used to investigate differences in FVC% predicted and DLCO% predicted at different time points. *P*-values < 0.05 were considered statistically significant.

## Results

### Baseline characteristics

Two hundred thirty-two (*n* = 232) patients were included in the analysis. Baseline characteristics are summarized in Table 1. Median age (95% CI) was 61.0 (58.0–63.0) years. Most patients were male (*n* = 160, 68.9%), while the most common comorbid condition was arterial hypertension (*n* = 88, 37.9%). Six patients (2.6%) had been vaccinated against COVID-19 prior hospitalization. One hundred seventy-eight (*n* = 178, 76.7%) patients reported history of hospitalization for COVID-19 and 54 (23.3%) patients were treated as outpatients. With regards to hospitalized patients, median value of the worst PaO_2_/FiO_2_ (95% CI) during hospitalization was 160.0 (130.2–180.0).

**TABLE 1:** Baseline characteristics of patients enrolled in the study.

Characteristics	(N%)
Total number of patients	232
Age (median% 95 CI)	61.0 (58.0–63.0)
Male/female	160 (68.9%)/72 (31.1%)
Current/ever/never smokers	30 (12.9%)/84 (36.3%)/118 (50.8%)
Hospitalization for COVID-19	178 (76.7%)
Vaccination against COVID-19	6 (2.6%)
Arterial hypertension	88 (37.9%)
Dyslipidemia	78 (33.6%)
Diabetes mellitus	55 (23.7%)
Thyroid disorders	24 (10.3%)
COPD	15 (6.4%)
Depression/anxiety	15 (6.4%)
Gastroesophageal reflux disease	15 (6.4%)
Asthma	13 (5.6%)
History of myocardial infarction	11 (4.7%)
Pre-existing ILD	8 (3.4%)
Cancer	8 (3.4%)
Atrial fibrillation	7 (3.0%)
Congestive heart failure	6 (2.5%)
Chronic kidney disease	5 (2.2%)
OSAS	3 (1.3%)
Pulmonary hypertension	2 (0.8%)

CI, confidence interval; COPD, chronic obstructive pulmonary disease; ILD, interstitial lung disease; OSAS, obstructive sleep apnea syndrome.

### Three-month follow-up

**FIGURE 1:**
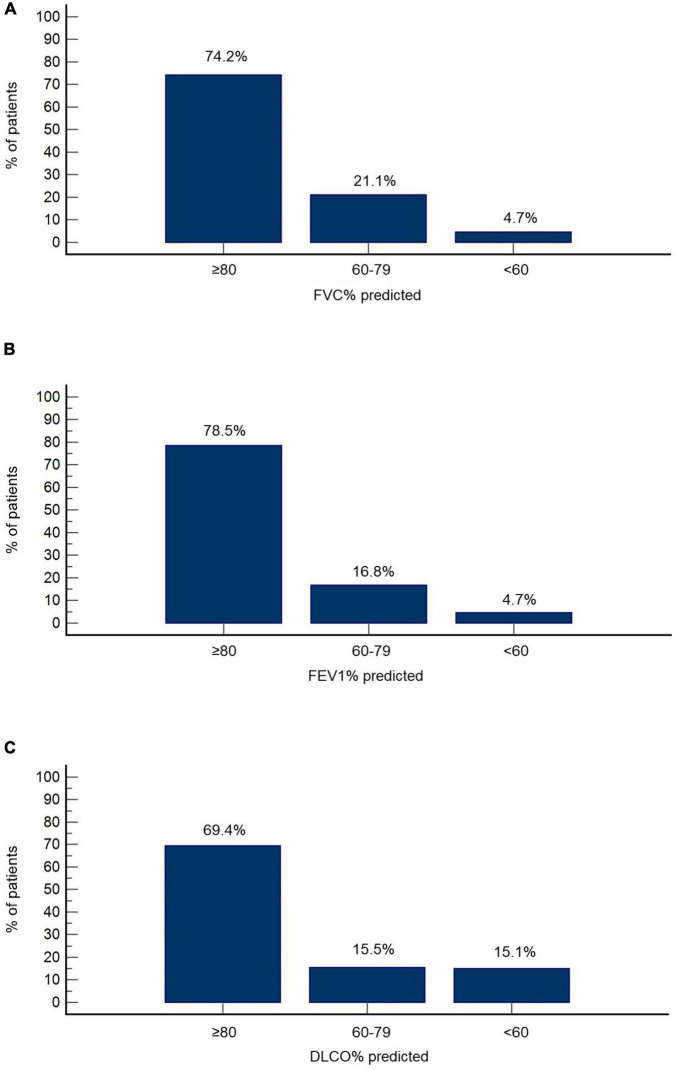
Functional features at the 3-month follow-up. Percentage of patients with reduced FVC% predicted **(A)**, FEV1% predicted **(B)**, and DLCO% predicted **(C)** is presented.

Functional and radiographic features at the 3-month follow-up are summarized in Table 2. FVC% predicted was ≥80, between 60 and 79 and <60 in 74.2% (*n* = 172), 21.1% (*n* = 49), and 4.7% (*n* = 11) of the cohort, respectively (Figure 1A). FEV1% predicted was ≥80 in 78.5% (*n* = 182), 60–79 in 16.8% (*n* = 39) and <60 in 4.7% (*n* = 11) of patients (Figure 1B). DLCO% predicted was ≥80, between 60 and 79 and <60 in 69.4% (*n* = 161), 15.5% (*n* = 36), and 15.1% (*n* = 35), respectively (Figure 1C).

**TABLE 2:** Functional and radiographic findings 3 months following hospitalization.

Parameter	Value
FVC% predicted (±SD)	89.3 (±18.8)
FEV1% predicted (±SD)	90.5 (±19.5)
DLCO% predicted (±SD)	85.8 (±27.3)
Hyperlucent% (±SD)	2.1 (±0.43)
Reticular% (±SD)	3.7 (±0.55)
Ground glass% (±SD)	8.9 (±1.0)
Honeycombing% (±SD)	0.36 (±0.22)

DLCO, diffusing capacity of lung for carbon monoxide; FEV1, forced expiratory volume in 1 s; FVC, forced vital capacity; SD, standard deviation.

**FIGURE 2:**
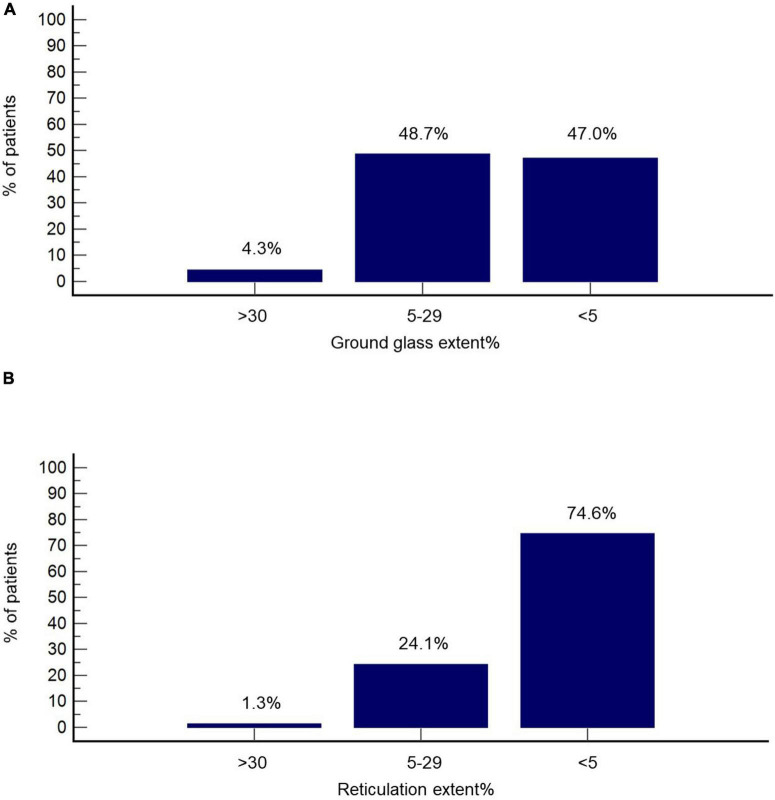
Radiographic features at the 3-month follow-up based on Imbio Lung Texture Analysis 2.1. Percentage of patients with% extent ground glass **(A)** and % extent reticulation **(B)** above 30, between 5 and 29, as well as below 5 is presented.

**FIGURE 3:**
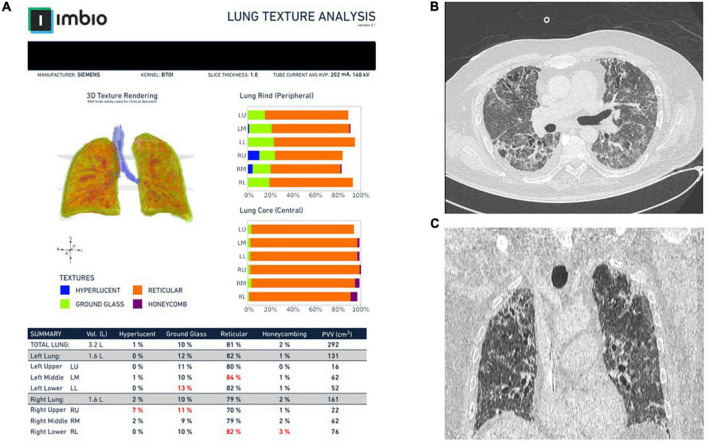
Representative images from Imbio Lung Texture Analysis 2.1 of a patient with post-COVID-19-ILD **(A)**. Notice that almost 80% of the lung parenchyma presents with reticular abnormalities that are inconspicuous (at least to that extent) to the bear eye of the operator **(B,C)**.

Imbio Lung Texture Analysis 2.1 demonstrated extent of ground glass opacities ≥30% in 4.3% of patients (*n* = 10), extent between 5 and 29% in 48.7% of patients (*n* = 113) and <5% in 47.0% of patients (*n* = 109) (Figure 2A). Accordingly, the extent of reticulation was ≥30, 5–29, and <5% in 1.3% (*n* = 3), 24.1% (*n* = 56), and 74.6% (*n* = 173) of the cohort, respectively (Figure 2B). Honeycombing was identified in 14 patients (6.0%). Representative images from Imbio Lung Texture Analysis 2.1 are presented in Figure 3.

### Post-COVID-19 interstitial lung disease

**FIGURE 4:**
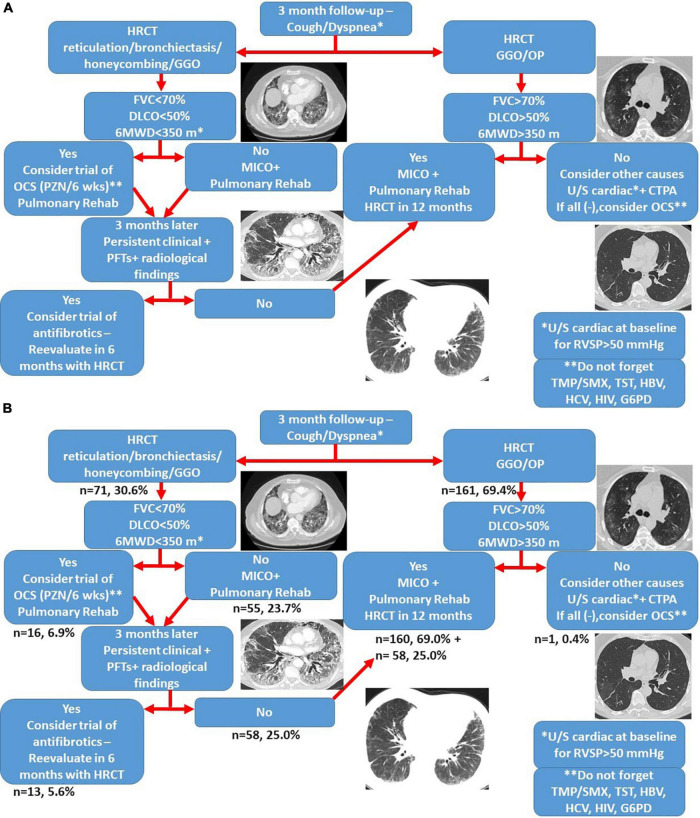
Treatment algorithm for patients with post-COVID-19 interstitial lung disease **(A)**. The proportion of patients that belongs to each group is shown in panel **(B)**. CTPA, computed tomography pulmonary angiogram; GGO, ground glass opacities; G6PD, glucose-6-phosphate dehydrogenase; HBV, hepatitis B virus; HCV, hepatitis C virus; HIV, human immunodeficiency virus; MICO, masterful inactivity with cat-like observation; OCS, oral corticosteroids; OP, organizing pneumonia; TMP/SMX, trimethoprim-sulfamethoxazole; TST, tuberculin skin test; 6MWD: 6 min walking distance.

Management of patients with post-COVID-19 interstitial lung disease was based on a common algorithm as shown in Figure 4A. Patients with reticulation >5% or honeycombing (*n* = 71, 30.6%) were meticulously evaluated at 6-months. Among them, patients with either of the following: FVC% predicted <70, DLCO% predicted <50 or 6-min walking distance <350 m (*n* = 16, 6.9%) had been offered a 6-week course of oral prednisolone with gradual tapering at the time point of the 3-month follow-up visit. The rest patients were meticulously evaluated as shown in Figure 4B and to this end none of them presented with disease progression.

**FIGURE 5:**
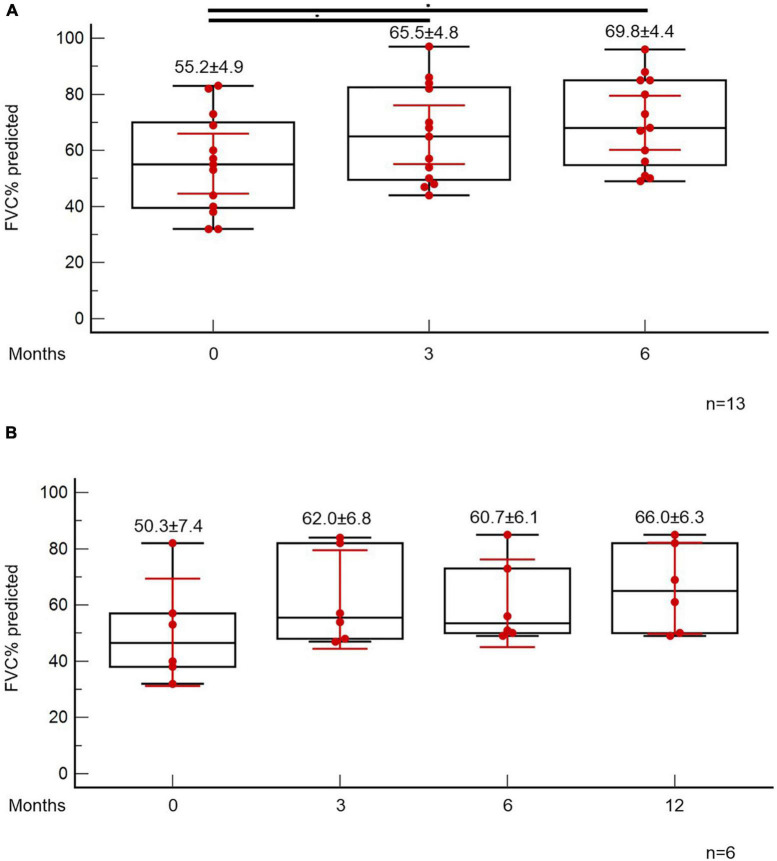
Patients with fibrotic-like changes and persistently decreased functional indices received antifibrotics. ANOVA repeated measures was used to investigate differences in FVC% predicted at different time points **(A)**. Subgroup analysis of patients with 1-year follow-up is presented in panel **(B)**.

**FIGURE 6:**
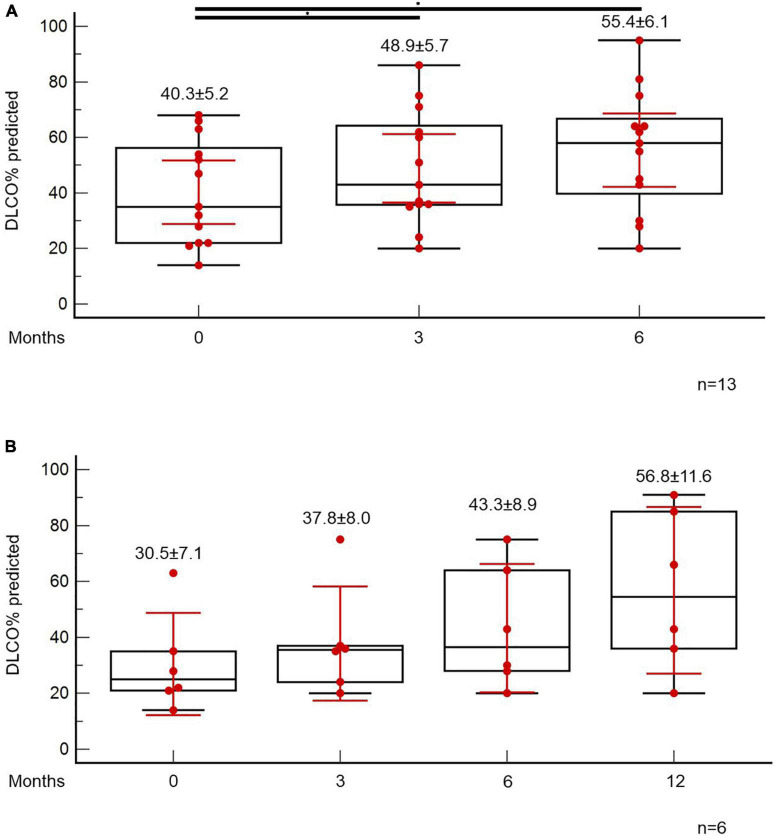
Patients with fibrotic-like changes and persistently decreased functional indices received antifibrotics. ANOVA repeated measures was used to investigate differences in DLCO% predicted at different time points **(A)**. Subgroup analysis of patients with 1-year follow-up is presented in panel **(B)**.

At the time point of the 6-month follow-up visit, 13 patients (5.6%) presented with evidence of both “fibrotic-like” abnormalities and persistently decreased functional indices as shown in Figure 4B. Of note, none of these patients was vaccinated against COVID-19 (0/13, 0%). In these patients, antifibrotics were implemented. Antifibrotics were chosen based on patients’ comorbidities and preferences following discussion for the potential adverse events of each compound (pirfenidone: 10, nintedanib: 3). A statistically significant improvement was observed with regards to ground glass opacities in the follow-up HRCT 6 months after initiation of antifibrotics [25.0 (95% CI: 6.7–27.9) vs. 5.0 (95% CI: 0.5–8.8), *p* = 0.04]. A trend for improvement of reticular opacities was also observed [8.0 (95% CI: 2.5–15.0) vs. 2.0 (95% CI: 1.0–8.9), *p* = 0.13]. Patients presented with an improvement (absolute change) of 10.3 (*p* = 0.01) and 14.6 (*p* = 0.01) in FVC% predicted at 3 and 6 months after the initiation of antifibrotic (Figure 5A). An absolute change of +8.6 (*p* = 0.0003) and +15.1 (*p* = 0.002) was observed with regards to DLCO% predicted (Figure 6A). Subgroup analysis of patients with 1-year follow-up is presented in Figures 5, 6B.

## Discussion

This is the first study in Caucasian population aiming to investigate post-COVID-19-ILD through a machine learning radiographic model. Our report presented in detail the functional and radiographic impact of this entity and validated previous evidence showing that a considerable proportion of patients is experiencing functional impairment several months following infection. Fibrotic lung disease was observed in a substantial minority of COVID-19 survivors. These patients might experience benefit from antifibrotic initiation at the time point that fibrotic-like changes are “immature.”

Our study exhibited a number of important attributes that should be presented. First, we evaluated disease extent through a machine learning model. Manual interpretation of radiographic abnormalities extent is hindered by variability, especially at centers with lack of expertise (17). Deep learning might be the key to overcome barriers for fast and massive radiological evaluation in an unbiased fashion. Deep learning could be deployed all over the world and provide homogeneous and accurate reporting (21–23). Second, we demonstrated that a short course of antifibrotics could significantly improve lung function. Third, based on the fact that the majority of our cohort was unvaccinated against COVID-19, we provided indirect evidence that vaccination against COVID-19 is not only the best way to contain the pandemic but also the best way to limit post-acute sequelae of COVID-19.

Percentage of patients with functional and radiographic impairment is in line from data of other countries (24–32). In the 3-month follow-up, almost 30% of this cohort presented with DLCO% predicted below 80. A recent, large study in China showed in the 6-month follow-up that DLCO% predicted was below 80 in 21% of patients that were not in need of oxygen supplementation and in 57% of hospitalized patients that presented with WHO ordinal scale of 5 or 6 (32). Reticulation >5% was observed in almost one out of four patients in this study. Other studies reported that fibrotic like changes ranged from 22.5 to 35% in the 6-month follow-up (26, 30). Importantly, we observed fibrotic lung disease and substantial functional impairment in 5.6% of this cohort. Previous studies reported significant functional deficit in almost 5% of COVID-19 survivors, while the prevalence of the so called post-COVID-19 interstitial lung damage was estimated between 6.5 and 8.3% (24, 25). Suggested risk factors for post-COVID-19 fibrotic lung disease have been male gender, increased age, increased body mass index, duration of hospitalization >17 days, disease severity, extent of baseline radiographic lesions, intensity of ventilatory support, persistent viremia and Epstein–Barr reactivation, diabetes mellitus, and presence of auto-antibodies (32–38).

With regards to treatment of post-COVID-19-ILD, a minority of our patients received oral corticosteroids. Percentage of treated patients in this cohort is comparable to a previously published large study reporting that corticosteroids were suggested initially in 35/837 COVID-19 survivors and finally prescribed in 3.6% (*n* = 30) of patients (24). Importantly, high doses of prednisolone (40 mg tapered) were not superior to lower doses (10 mg tapered) in patients with post-COVID-19-ILD (39). In the context of “fibrotic-like” changes, sequelae consistent with “fibrotic-like” parenchymal lung disease has been observed following COVID-19 infection in a minority of patients and importantly these changes seem to lack of resolution in a considerable proportion of cases (40); yet, estimates must be interpreted cautiously due to substantial heterogeneity, differences in study casemix, and baseline severity among studies (41). Antifibrotics were prescribed in a substantial minority of this cohort. Pirfenidone was administered more often, as a considerable proportion of patients had history of pulmonary embolism following COVID-19 infection or was receiving anticoagulants due to chronic heart disease. While high quality trials aiming to address their role in post-COVID-19 interstitial lung disease are greatly anticipated (42–44), observational studies have suggested that antifibrotics might confer benefit (45, 46) with regards to radiologic improvement and time to recovery in patients with sustained and extensive fibrotic changes. The concept of antifibrotics implementation in a minority of patients at the time point that fibrotic-like changes are “immature” deserves further investigation (47). It is a matter of ongoing debate if all abnormalities characterized as “fibrotic-like” can reliably indicate irreversible disease in a post-Acute Respiratory Distress Syndrome setting (48, 49). Eminent fibrotic-like changes and architectural distortion on HRCT is not necessarily synonymous with irreversible lung scarring, particularly in the context of a post-infectious syndrome (50). Similarly, bronchiectatic-like lesions during the acute phase of a respiratory infection may not necessarily represent irreversible and permanent enlargement of the airways but may largely resolve following resolution of the infection. Even if “fibrotic-like” changes on HRCT represent histologic fibrosis in a subgroup of cases, remodeling and regression of “immature” fibrosis represents an anticipated phenomenon following severe acute lung injury (49–51). In such cases, antifibrotics might be beneficial. Machine learning models might be helpful for the appropriate classification and selection of patients likely to benefit from antifibrotics as they may quantify in an unbiased and accurate fashion the extent and the type of lesions.

Our trial has some limitations. First, our report has the inherent weaknesses of a retrospective study. Second, follow-up period of patients under antifibrotics is relatively short given that post-COVID-19-ILD is a new entity; yet, even in this short period, our study provided evidence that antifibrotics might confer significant benefit if applied early during the post-COVID-19 syndrome when fibrotic-like changes appear to be “immature.” Third, a major limitation is that this cohort has not undergone HRCT prior SARS-CoV-2 infection to compare pre and post-infection radiographic abnormalities and identify a subset of patients with pre-existing ILD patterns. Nonetheless, the percentage of patients with functional and radiographic impairment is similar to other published reports and importantly our aim was to highlight the need of early detection and management of interstitial lung disease. Moreover, based on current guidelines, HRCT during infection was not performed in all cases, while PFTs could not be performed at the acute phase due to safety considerations. Thus, we could not compare radiographic and functional status between the acute phase and the 3-month follow-up. Finally, based on the fact we used a common algorithm and treated similarly all cases of same severity, we could not compare patients that receive antifibrotics with other patients of same phenotype that didn’t. Besides, this was not a randomized-controlled trial. This was a real-life study aiming to present outcomes following management with a common algorithm and using a highly novel machine learning radiographic model.

Collectively, post-COVID-19-ILD represents an emerging entity. Given that a considerable proportion of infected individuals has functional impairment several months after infection, screening 3 months following infection is encouraged, especially for severe cases that have been hospitalized for prolonged periods. On the other hand, post-COVID-19 HRCT screening may confer several other benefits, including identification of incidental lung cancer lesions, considering that the majority of patients screened for post-COVID-19 present with several risk factors for lung cancer, such as age, smoking status and comorbidities, and including chronic respiratory diseases. Current evidence does not support irrational use of corticosteroids; yet, short-term corticosteroids might confer benefit to specific patients with organizing pneumonia, functional impairment and persistent clinical symptoms without compromising patients’ immune status. Finally, a minority of these patients with persistent fibrotic lung lesions and functional disability, despite steroid treatment, could benefit from antifibrotic therapy, if applied early on during disease clinical course. Machine learning radiographic models might have a cardinal role for precise, unbiased and quantitative radiographic evaluation which may guide therapeutic decisions.

## Data availability statement

The original contributions presented in this study are included in the article/supplementary material, further inquiries can be directed to the corresponding author.

## Ethics statement

The studies involving human participants were reviewed and approved by the International Conference on Harmonization E6 guidelines for Good Clinical Practice, the Declaration of Helsinki and the local regulation. Written informed consent for participation was not required for this study in accordance with the national legislation and the institutional requirements.

## Author contributions

TK and VS: conception and design of the work, acquisition, analysis and interpretation of data, and drafting the work and revising it critically for important intellectual content. MK, PT, VG, IP, EB, IT, IL, OP, EZ, EA, AP, EM, FS, SM, SC, GH, KD, NT, NS, and KA: contributions to the acquisition of data and critical revision for important intellectual content. AT: conception and design of the work, acquisition, analysis and interpretation of data, drafting the work and revising it critically for important intellectual content, formal analysis, and project administration and supervision. All authors final approval for publication of the content, agreement to be accountable for all aspects of the work in ensuring that questions related to the accuracy or integrity of any part of the work are appropriately investigated and resolved.
