# Corrigendum: Post-COVID-19 interstitial lung disease: Insights from a machine learning radiographic model

**DOI:** 10.3389/fmed.2023.1194925

**Published:** 2023-04-13

**Authors:** Theodoros Karampitsakos, Vasilina Sotiropoulou, Matthaios Katsaras, Panagiota Tsiri, Vasiliki E. Georgakopoulou, Ilias C. Papanikolaou, Eleni Bibaki, Ioannis Tomos, Irini Lambiri, Ourania Papaioannou, Eirini Zarkadi, Emmanouil Antonakis, Aggeliki Pandi, Elli Malakounidou, Fotios Sampsonas, Sotiria Makrodimitri, Serafeim Chrysikos, Georgios Hillas, Katerina Dimakou, Nikolaos Tzanakis, Nikolaos V. Sipsas, Katerina Antoniou, Argyris Tzouvelekis

**Affiliations:** ^1^Department of Respiratory Medicine, University General Hospital of Patras, Patras, Greece; ^2^Department of Infectious Diseases-COVID-19 Unit, Laiko General Hospital, Athens, Greece; ^3^Department of Respiratory Medicine, Corfu General Hospital, Corfu, Greece; ^4^Laboratory of Molecular and Cellular Pneumonology, Department of Thoracic Medicine, Medical School, University of Crete, Heraklion, Greece; ^5^5th Department of Respiratory Medicine, Hospital for Thoracic Diseases, ‘SOTIRIA', Athens, Greece; ^6^Medical School, National and Kapodistrian University of Athens, Zografou, Greece

**Keywords:** post-COVID-19, long COVID, interstitial lung disease, antifibrotics, machine learning

In the published article, the Funding statement was not reported. The correct Funding statement appears below.

